# Influence of noise manipulation on retention in a simulated ICU ward round: an experimental pilot study

**DOI:** 10.1186/s40635-022-00430-1

**Published:** 2022-01-28

**Authors:** Katja Erne, Samuel E. J. Knobel, Aileen C. Naef, Stephan M. Gerber, Tim Fischer, Fred W. Mast, Joerg C. Schefold, Bjoern Zante, Tobias Nef, Marie-Madlen Jeitziner

**Affiliations:** 1 Gerontechnology and Rehabilitation Group, University of Bern, Murtenstrasse 50, 3008 Bern, Switzerland; 2 Department of Intensive Care Medicine, Inselspital, Bern University Hospital, University of Bern, Bern, Switzerland; 3 Department of Cognitive Psychology, Perception and Research Methods, University of Bern, Bern, Switzerland; 4 ARTORG Center for Biomedical Engineering Research, University of Bern, Bern, Switzerland; 5 Department of Neurology, Inselspital, Bern University Hospital, University of Bern, Bern, Switzerland; 6 Institute of Nursing Science (INS), Department of Public Health (DPH), Faculty of Medicine, University of Basel, Basel, Switzerland

**Keywords:** Intensive Care Unit, Noise, Ward round, Retention, Pilot study

## Abstract

**Background:**

Noise exposure leads to a reduction in cognitive abilities in diverse settings, however, only limited data exist examining the effects of environmental ICU noise on the cognitive performance of ICU professionals. A frequently occurring and demanding retention task in ICUs are ward rounds, which are considered key for the provision of medical care. Here, we investigate the influence of noise on information retention in a simulated ward round.

**Methods:**

ICU professionals were exposed to a recorded, ICU ward round, simulated partly with and partly without environmental ICU noise. Ward rounds were followed by specific questions about previously provided information.

**Results:**

56 ICU professionals (aged 26–59 years) were included. A logistic mixed model showed a reduction of 27% (*P* < 0.001) in the ward round test performance when participants were exposed to environmental ICU noise. Furthermore, advanced age was associated with reduced retention (− 28%, *P* < 0.001), questions containing important information performed better (+ 36%, *P* < 0.001), and higher stress led to better performance in retention (+ 24%, *P* = 0.01).

**Conclusions:**

Our data showed a considerable negative influence of environmental ICU noise during a simulated ward round. Therefore, reduction of environmental ICU noise is recommended. The influence of additional factors, including stress, priorities, and demographic factors should be pursued in subsequent investigations.

**Supplementary Information:**

The online version contains supplementary material available at 10.1186/s40635-022-00430-1.

## Background

Intensive Care Unit (ICU) professionals are exposed to a lot of noise in their ICU work environment [1, 2]. This raises concerns as noise has been found to have a negative impact on cognitive performance in other occupational settings [3]. However, at this time, there is a lack of knowledge about the impact of increased noise exposure on the work performance of ICU professionals.

In the ICU, examples of the most common sound sources are equipment (e.g. monitor or ventilator alarms), patient care (e.g. patient transfer), patients themselves (e.g. coughing), the immediate work environment (e.g. staff conversations), and general background noise [1]. When combined, ICU sound levels can reach average decibel levels as high as 70 dBA (66 + 6.1dBA) [4]. However, the sound levels within the ICU can vary over time [4], for example depending on proximity to the patient or with the occurrence of group discussions [5]. Specific to the ICU, and in the context of this study, the sound environment (i.e. patient alarms, coughing, yelling, preparing medication, and equipment sounds), is defined as noise when there is a negative perception associated to it [6, 7]. This is the case for ICU professionals, as they perceive the aforementioned sounds in their environment as being negative [2]. Heightened noise levels in ICUs have subjectively been found to induce annoyance and stress, decrease overall well-being, and reduce work performance [2]. However, as noise is subjective it may in certain circumstances not be perceived negatively, for example for some people the phone ringing may be associated with a positive reaction or memory.

The impact of background noise in a non-hospital setting has been widely discussed in the literature and reduced performance in cognitive functions has been shown, such as in working memory [8], recall [9], and attention [3]. Additionally, further factors, such as work experience [10, 11], stress [12, 13], age [14], and motivation [10] may also influence cognitive functions or work performance. Specifically, in the hospital setting, limited studies have investigated the noise effect on cognitive performance. One study by Murthy et al., that did look at this, found that medical professionals had lower performance on short-term memory and mental efficiency when exposed to operating room background noise [15]. Furthermore, higher error rates were shown in an anaesthesiology setting when exposed to environmental noise [16]. As the presence of environmental noise distracts medical professionals, especially during cognitively demanding tasks [17], they might be particularly affected by environmental ICU noise.

The effect of noise could be especially important during ICU ward rounds, which are used to communicate between shifts [18]. Ward rounds are known to be cognitively demanding as they contain complex content [19] and have the potential to directly impact ICU care [20]. Specifically, ward rounds involve multiple individuals gathering around the patient bed to share information amongst themselves. This differs from standard patient care around the bed, like delivering medication to the patient, which is usually done by a single nurse. We, therefore, embarked to investigate the influence of environmental ICU noise on the retention ability of ICU professionals in a simulated ICU ward round. Other influencing variables, such as age, stress, and motivation were also analysed.

## Methods

The aim of the simulated ward round was to assess the influence of ICU noise on the retention ability of ICU professionals, in an experimental setting. Potential influencing variables were also included for a more precise influence analysis of the retention ability after ward rounds.

### Participants

Participants were ICU professionals, namely nurses and physicians, working in the Department of Intensive Care Medicine at the University Hospital Bern in Switzerland. They were invited via word-of-mouth during their working shift. Professionals were excluded if any of the following criteria were met, age under 18 years, pre-existing known neurological disorder or auditory impairment, or use of prescribed medication that could alter the level of alertness. Participation was voluntary and all participants signed a written informed consent. The study was approved by the local ethics committee at the University of Bern (No.: 2020-04-00,009). The study was performed in adherence to the Declaration of Helsinki.

### Procedure

This pilot study took place in a quiet room in the hospital, where a maximum of two ICU professionals could participate at a time. When there was more than one participant at a time, they sat with their backs to each other while wearing noise-cancelling over-ear headphones. Each participant listened to eight different audio files (i.e. eight patient cases presented in a single ICU ward round, for detailed content see Additional file 1: S3 and for more audio details see section Audio Files) in a randomised order. Participants were instructed to memorise the content of the ward round as they would during real-life ward rounds. Participants were allowed to take written notes of any information, as if in real-life ward rounds.

All the cases presented in the ward round had two versions, one version included only the speech of the ward round, and the second version included simulated ICU noise in addition to the speech (Speech Condition vs. Noise Condition). The participants then listened to half of the cases in the Speech Condition and the other half in the Noise Condition. The starting condition and the order in which the eight patient cases were presented to the participants were randomised, while the conditions (i.e. Speech Condition vs Noise Condition) were presented alternately. Directly after listening to the eight audio files, participants’ demographics were collected, and the ward round test (for details see section Questionnaires and Additional file 1: S3), noise sensitivity, and individual variables (e.g. motivation, concentration) were assessed via questionnaires (Additional file 1: S2 and S3).

### Audio files

Each audio file contained a simulated ICU ward round case, designed and recorded by an ICU physician (Additional file 1: S1). Audio files consisted of 301–385 words (*M* = 341.4, SD = 27.6) and were played at a weighted sound pressure level of 70 dB SPL. In the Noise Condition simulated ICU noise was added. The simulated noise was manually constructed by adding different ICU sound sources, such as alarms and pagers, to ICU background noise recorded in a real ICU environment. Due to the limited effect of alarms on overall sound levels [21], the audio files are based mainly on unintelligible background conversations. The frequency range of simulated noise files were between 50 Hz and 22 kHz, with peaks between 2.2 and 3.4 kHz that resulted from the pagers. Each simulated noise file contained six peaks. The noise was played with a signal-to-noise ratio of − 5 dB SPL so that the speaking voice could be understood in all cases (Additional file 1: S4).

### Questionnaires

A self-constructed test of each ward round was assessed using an eight-item free recall questionnaire (Ward round test, Additional file 1: S3). During the creation of the test, two ICU professionals rated the importance of each question (“1 not important” to “5 very important”) and all questions with a mean rating below 3 were replaced by new questions with higher ratings of importance. The final questions at the end, therefore, included only questions rated as important (rating mean ≥ 3), such as current medication, Glasgow Coma Score, reason for being at the ICU, and the state of affairs with the relatives.

Additionally, the Weinstein Noise Sensitivity-Scale (WNS) [22] which applies a six-point Likert scale (1 “strongly disagree” to 6 “strongly agree”), to rate the individual noise sensitivity in different noise situations was used. Questions looking at the individual participant variables (e.g. concentration, motivation) were based on self-reports of the participants, they included direct questions such as “How motivated were you to perform well during the study?” with an answer scale of 0 “very low” to 100 “very high” (for full list of participant variables see Additional file 1: S2).

### Statistical analysis

Sample size and statistical power was investigated using Monte Carlo simulations. Five ICU experts tried the ward round test, based on these results, we assumed that if noise was present (Noise Condition) a correct response rate of 50% would be obtained. An improvement of 10% was assumed to be clinically relevant [23]. With these assumptions, a sample size of 50 subjects and an alpha of 5% would provide a statistical power of 89%.

The effect of the Noise versus Speech Conditions in the audio files on the probability of a correct answer was calculated using a logistic mixed model with the controlled random effects of the participant number, patient, and question number. The influencing factors included presence of ICU noise, age, importance of question, stress, monthly working hours, working hours before study participation, noise sensitivity, subjective hearing performance, concentration, concentration in noise environments, energy, motivation, and notes habit. These factors were included as fixed factors in the model calculation.

Fisher’s exact test was used to assess if the distribution of the cases (how many times a case was answered in a certain condition) was significantly different from a random distribution [24]. All calculations were made using the statistical program R-Studio [25].

## Results

A total of 56 ICU professionals (*n* = 12 physicians, *n* = 44 nursing professionals) participated in the study between May and June 2020. Mean age was 38.4 ± 8.40 years [26; 59] and 17 were male, 38 were female, and 1 was not specified (Table 1).

**Table 1:** Participant demographics

Detailed demographics	
Characteristic	Value
Work experience as ICU professional, years	9.28 ± 8.46, [0.17; 36]
Participants working before participating in the study	40 (71.4%)
Mean working time before study participation, hours	4.79 ± 2.29, [1; 9]
Preferred working environment - Quiet environment - No preference - Noisy environment	46 (82.1%) 8 (14.3%) 2 (3.6%)
Subjective hearing performance 0 “very bad” to 100 “very good”	60.7 ± 17.0

Information was collected after listening to the ward rounds but prior to retention testing (ward round test), mean ± SD, [min; max], or number of participants *n* (%) are given

### Correct answers per case

Descriptive analyses showed the percentage of the correct responses given in the ward round test per case. Percent correct answers in the ward round test showed higher retention performance in the Speech Condition in six of the eight patient cases presented during the ward round (Table 2). Due to the randomisation of the order of the cases, the two conditions came up with different frequencies. Nevertheless, according to Fisher’s exact test for data count there was no case-condition that was significantly more or less frequent than others (*P* = 0.55).

**Table 2:** Percent correct answers in the ward round test

Case	Noise condition [%]	Speech condition [%]
1	52 (*n* = 33)	58 (*n* = 23)
2	54 (*n* = 27)	70 (*n* = 29)
3	52 (*n* = 28)	56 (*n* = 28)
4	69 (*n* = 29)	68 (*n* = 27)
5	57 (*n* = 24)	69 (*n* = 32)
6	61 (*n* = 32)	59 (*n* = 24)
7	57 (*n* = 23)	70 (*n* = 33)
8	55 (*n* = 28)	60 (*n* = 28)

Displays correct answers given by the participants (n) per condition and case

### Noise-effect on influencing variables

In the mixed logistic regression analysis, the Noise Condition led to a decrease of 27% (*P* < 0.001, Table 3) in the ward round test performance. Moreover, a negative effect for higher age was observed (− 28%, *P* < 0.001), more important questions performed better (+ 36%, *P* < 0.001), and higher perceived stress in the past week led to better performance in the ward round test (+ 24%, *P* = 0.01).

**Table 3:** Complete performance estimate-based influencing factors

	Odds ratio	95% CI	*z* value	*P* value
Noise	0.73	0.63–0.84	− 4.31	0.00
Age	0.72	0.61–0.85	− 3.99	0.00
Importance of question	1.36	1.26 − 1.46	7.80	0.00
Stress	1.24	1.04–1.47	2.47	0.01
Monthly working hours	0.99	0.82–1.19	− 0.12	0.91
Working hours before study participation	0.88	0.76–1.03	− 1.57	0.12
Noise sensitivity (WNS)	0.90	0.75–1.08	− 1.10	0.27
Subjective hearing performance	0.95	0.82–1.11	− 0.62	0.54
Concentration	0.88	0.72–1.09	− 1.16	0.25
Concentration in noise environments	1.00	0.82–1.21	− 0.05	0.96
Energy	0.99	0.83–1.15	− 0.06	0.95
Motivation	0.97	0.83–1.15	− 0.32	0.75
Taking notes as in real ward rounds	0.77	0.57–1.05	− 1.63	0.10

*WNS* Weinstein noise sensitivity-scale

## Discussion

Our study showed a considerable decline in performance in the ICU ward round test when background noise was present. Moreover, with advanced age the participants showed more errors in the ward round test, and ward round test questions rated as more important were answered correctly more often. Lastly, more stressed participants had higher test results in the ward round test. The self-perceived noise sensitivity seemed not to affect the ICU ward round test performance.

The ICU professionals in this study showed a 27% reduction in retention performance in the ward round test during the Noise Condition. This is consistent with literature from outside the hospital, regarding the impact of environmental noise on memory retrieval performance [9, 26, 27]. Additionally, the result is supported by findings which show that noise leads to higher error rates and decreases mental efficiency and short-term memory in a medical context [15, 16]. Unfortunately, many of the past studies did not measure the performance with real daily work exercises. Therefore, we deliberately aimed to mimic a real ICU ward round, including allowing participants’ to take notes. Note taking is known to have a positive effect on deeper information processing [28] and should, therefore, lead to better results in retention tests. However, as the participants performed worse in the ward round test, even if they took notes and used them to answer the task-related questions, this supports the finding that noise has a strong negative effect on retention. As the performance of medical professionals is essential for good medical care [20], noise reduction during ward rounds is recommended to increase patient safety.

Our study showed a negative effect of age on the ward round test performance. This is in line with literature which confirms that older people make more long-term selective attention and concentration errors [29]. Moreover, age-related functional brain changes may lead to a reduction of different memory functions [30–32]. Therefore, our findings regarding age are not surprising, however, other aspects, such as compensating strategies [33], or individual variables such as test anxiety [34] may be related to performance in age and were not taken into account. Another possible explanation might be the reduced capabilities of distinguishing background sounds with increasing age [35]. As hearing performance was not objectively assessed in this study, the question of age-related performance may be an interesting topic for future investigation.

This study only included questions subjectively rated as important. However, from these questions, those with a higher score of importance were more often answered correctly by the ICU professionals. This may occur due to learning effects due to repeating test questions [36]. ICU professionals respond daily to routine questions, which they inevitably have to be able to answer after ward rounds during their work routine. In this way, certain questions may have been answered correctly more often due to their importance and appearance in daily, real-life, ward rounds.

Our study also showed better retention results in ICU professionals experiencing more stress in the past seven days. The effect of stress on performance remains conflicting in the literature [37]. On the one hand, an immediate stress reaction induced via glucocorticoids can enhance memory retrieval and consolidation [12], however on the other hand, studies have also found impaired memory retrieval under stress [13]. Therefore, further research is needed to understand the full effect of stress on the retrieval performance of ICU professionals.

The self-reported noise sensitivity of the participants showed no effect on the ward round test, which is not in line with most of the literature [38, 39]. A possible explanation for this discrepancy might be that ICU staff becomes less sensitive to ICU alarms over time [40], which may lead to less ICU background noise-induced distraction. Some literature, such as Ljungberg and Neely support our finding, as they found that noise sensitivity was not a relevant factor in an attention task [41]. Another reason might be that our study did not had enough power to find an effect of self-reported noise sensitivity. Therefore, the relevance of noise sensitivity of cognitive functions remains controversial and needs further investigations with studies where the sample sizes are powered to this hypothesis.

Our study had some limitations that include the monocentric study design which may limit external validity [42]. However, most hospitals do provide information and lead discussions at the ICU bed, which may be influenced by noise. Therefore, this is an organisational aspect to be considered in the structural layout of future ICUs, in order to permit ward rounds to be conducted at a distance from the patient bed. Additionally, hospitals which conduct ward rounds in this way could consider adapting how they manage the transfer of information.

Because noise is a factor which, from the hospital side, can be adjusted, this pilot study focused on the noise influence on memory retrieval of ICU professionals. However, we measured superficially other aspects (screening), which were found to be important, too. In further studies, however, a deeper focus on these aspects is necessary. Especially, the inclusion of other memory tasks might lead to complementary results.

Moreover, due to the investigatory nature of a pilot study ICU professionals were grouped in this first analysis, however, future work would need to differentiate between these two groups.

Finally, absence of real patients in the experimental setting could be a limitation. On the one hand, the presence of patients may be important, because they could support the memorisation of information as it is being received both visually and auditory [43], on the other hand, distractions during the ward round because of interaction with the patient, might also have a negative impact [44]. Therefore, the retention in real ward rounds should be investigated in future studies.

## Conclusions

Environmental noise in the ICU during ward rounds negatively influences retention of ICU professionals. Our data lend to the fact that reducing noise, e.g. by means of targeted interventions, should be investigated. Ideally these interventions will target age, stress, and the influence of important questions.

## Take home message

1. Environmental ICU noise reduced retention in ICU professionals by about 27%.
2. Younger age, importance of information, and stress level positively influenced the retention performance of ICU professionals.

## Supplementary Information

**Additional file 1. Part 1**. Cases.** Part 2**. Full Demographic Questionnaire.** Part 3**. Ward round questionnaire.** Part 4**. Audio files.

## Data Availability

The dataset used and analysed during the current study are available on reasonable request.

## Abbreviations

ICU: Intensive Care Unit
dBA: Decibel (A-weighted)
dB SPL: Decibel sound pressure level
WNS: Weinstein Noise Sensitivity-Scale
