# Supplementary material for: Influence of noise manipulation on retention in a simulated ICU ward round: an experimental pilot study
Source: Intensive Care Med Exp. 2022 Jan 28;10:3. doi: 10.1186/s40635-022-00430-1 (PMC8799802; doi:10.1186/s40635-022-00430-1)
Supplement: Supplementary file 1 — Additional file 1. Part 1. Cases. Part 2. Full Demographic Questionnaire. Part 3. Ward round questionnaire. Part 4. Audio files. [file 40635_2022_430_MOESM1_ESM.docx]

# Additional File

## Part 1: Cases

*German*

*Case 1*

Hier liegt Herr Reto Stämpfli, geboren am 30.11.51; 1,78m groß, 95 Kilo schwer; hat vor 10 Tagen einen Autounfall gehabt mit schwerem Schädel-Hirn-Trauma, einer intrazerebralen Blutung, initial hatte er GCS 9, multiple Gesichtsschädelfrakturen, sowie eine BWK 4/5 Fraktur, die instabil ist; beidseits Unterschenkelfrakturen, sowie ein stumpfes Bauchtrauma mit Milzlazeration Grad 3 und Nierenlazeration Grad 2; ein Thoraxtrauma mit Rippenserien-Frakturen rechts. Initial zeigte er sich mit einem erhöhten Hirndruck mit maximal 30. Daraufhin wurde er dekompressiv kraniektomiert, rechts, am zweiten Tag. Danach hatten wir jedoch weiter Hirndruck-Problematiken, daher zunächst kein Aufwachversuch. Jetzt sind die Hirndrücke unter Kontrolle; im Aufwachversuch hat er weiterhin GCS von 9. Die Versorgung des Gesichtsschädels soll konservativ erfolgen, ebenso die Milz- und Nierenlazeration. Im Verlauf konnte die Therapie mit den kreislaufunterstützenden Medikamenten beendet werden. Am 6. Tag kam es zu einer zunehmenden Verschlechterung der Beatmungssituation; Im Röntgenbild zeigten sich Verschattungen beidseits. Bei Anstieg der Entzündungszeichen und Fieber wurde mit der antibiotischen Therapie Augmentin begonnen. Das Sekret, was wir absaugen konnten, war trüblich-gelb, TBS ist abgenommen worden und im Thoraxsonographie zeigte sich kein Pleuraerguss. Die aktuellen Beatmungsstellungen sind PEEP-8, FiO2= 0.5. Im weiteren Verlauf kam es zu einer erneuten Kreislaufinsuffizienz im Sinne eines septischen Schocks bei postulierter Lungenentzündung. Hier wurde die antibiotische Therapie auf Tazobac eskaliert. Darunter sind die Entzündungsparameter rückläufig, jedoch stiegen die Retentionsparameter an und es kam zum Nierenversagen. Bei steigendem Harnstoff auf 34 und Anurie, sowie Hyperkaliämie folgte jetzt seit drei Tagen das kontinuierliche Nierenersatzverfahren. Aktuell mit einem Entzug von 200ml je Stunde. Die Kalorie von gestern Nacht zeigt 2200 Kilokalorien an; die enterale Ernährung wird entsprechend gesteigert.

Aufträge für den nächsten Dienst: Negativbilanzierung, Abwarten der mikrobiologischen Diagnostik der Lungenentzündung und gegebenenfalls Beendigung der antibiotischen Therapie.Des Weiteren muss das Gespräch mit der Lebensgefährtin, Frau Gisela Roter, über das weitere Prozedere geführt werden. Hier soll die Einwilligung zur Tracheotomie eingeholt werden, wenn der GCS weiter nicht besser wird; und die Rücksprache mit den Orthopäden, wann das BWK versorgt werden soll.

In der weiteren Anamnese von Herrn Stämpfli gibt es einen arteriellen Hypertonus, Status nach Blinddarmentfernung 1970, Status nach Katarakt-OP 2016 links, sowie Status nach CVI 2017 mit einem leichten Hemi auf der rechten Seite

*Case 2*

Hier liegt Frau Rosi Müller, geboren am 25.04.1938. Sie ist 1.65 groß, wiegt 78 kilo. Sie ist vor elf Tagen von einer Treppe gestürzt, hat sich dabei ein Schädel-Hirn-Trauma zugezogen mit intrazerebraler Blutung. Initial betrug der GCS 6. Sie wurde vor Ort intubiert, hat eine komplexe Beckenfraktur erlitten, sowie eine Rippenserienfraktur links, 2-8 ventral. Sie hat eine Unterschenkelfraktur auf der linken Seite, sowie eine Humerusfraktur auf der linken Seite. Bei Instabilität wurde im Schockraum ein Fixateur Extern am Becken angelegt. Im Aufwachversuch zeigte sie sich zunächst nicht adäquat, mit einem GCS von 8. Im Verlaufs-CT vom Kopf zeigte sich dann ein Aufblühen der Kontusionsblutung. Daraufhin erfolgte die Anlage einer EVD. Die ist jetzt auf 15 offen.

Am sechsten Tag bei stabilen Hirndruckwerten erfolgte die operative Versorgung des Beckens durch die Orthopäden. Im Aufwachversuch zeigte sie ein GCS von 13 und konnte anschließend exturbiert werden. Jedoch musste sie reinturbiert werden, bei insuffizienter Atemmechanik und viel Sekret. Das aktuelle Röntgen-Thoraxbild zeigt eine Atelektase auf der linken Seite. Hier ist eine Bronchoskopie erfolgt. Es konnte viel glasiges, zähes Sekret aus dem Mittellappen entfernt werden. Danach war die Beatmungssituation deutlich besser und das FiO2 konnte von 0.5 auf 0.35 zurückgenommen werden. Der PEEP beträgt 5. Dann kam es zur Entwicklung eines Vorhofflimmerns, darunter wurde sie hämodynamisch instabil und wir haben mit Noradrenalin begonnen. Die zentralvenöse Sauerstoffsättigung betrug 45. Daraufhin haben wir ein Pulmonaliskatheter eingeschwemmt. Hier betrug die gemischtvenöse Sättigung 45, der Cardiacindex 1.8, sodass wir mit Dobutamin angefangen haben. Darunter verbesserte sich die Hämodynamik und mittels Kardioversion konnte ein Sinusrhythmus etabliert werden und das Dobutamin konnte reduziert werden. Im TTE zeigte sich eine EF von 45%.

Die Aufträge für den nächsten Dienst: Dobutamin und Noradrenalin ausschleichen, Rücksprache mit den Thoraxchirurgen und Evaluation des instabilen Thorax und ggf. Thoraxstabilisierung. Dann muss noch das Gespräch mit dem Lebenspartner, Herrn Reto Schüppach erfolgen, über das weitere Prozedere.

In der weiteren Anamnese von Frau Müller hat sie eine KHK, ein paroxysmales Vorhofflimmern, Status nach Mamma-Ca, Hüftendoprothese vor sechs Jahren auf der linken Seite, sowie eine Hypothyreose, die mit 150 Mikrogramm Euthyrox substituiert ist.

*Case 3*

Hier liegt Frau Marie Egli, geboren am 16.07.1946, sie ist 1.65 groß und wiegt 69 Kilo. Sie hat vor vier Tagen eine Magenperforation erlitten und daraufhin eine Notfalllaparatomie bekommen, bei beginnender Sepsis. Hier wurde ihr eine Magenteilresektion zugeführt. Weiterhin, beim septischen Schock, hat sie hochdosiert Noradrenalin gehabt und acht Liter Positivbilanz. Die antibiotische Therapie erfolgt mit Cefepime und Metronidazol. In der mikrobiologischen Diagnostik hat sich bis dato kein Nachweis von Keimen ergeben. Im Verlauf kam es zur weiteren Kreislaufinstabilität, sodass wir ein pulmonales Katheter eingelegt haben. Hier zeigte sich ein Herzindex von 1.5, sowie ein ZVD von 15. Echokardiographisch zeigte sich eine deutlich eingeschränkte Pumpfunktion mit einer LVEF von 30%, sodass wir mit Dobutamin begonnen haben. Darunter verbesserte sich die Kreislaufsituation. CardiacIndex war 1.9, ZVD 10, gemischtvenöse Sauerstoffsättigung 65% und das Laktat kam von 8.6 auf 3.9 mmol/l zurück. Klinisch fiel dann ein deutlich gespanntes Abdomen auf, sodass die Viszeralchirurgen eine erneute Laparatomie durchführten. Hier zeigte sich eine nekrotische Magenschleimhaut, sodass eine komplette Magenteilresektion durchgeführt wurde. Die Patientin ist jetzt zwei Stunden aus dem OP. Das Laktat ist jetzt 2.5, Dobutamin ist auf gleichbleibender Dosierung, Noradrenalin konnte jedoch deutlich reduziert werden. Der dritte Look ist in 48h geplant.

Die Aufträge für die nächste Schicht sind: Wenn möglich das Noradrenalin reduzieren, bei warmer Peripherie und erhaltener Diurese beginnen mit Negativbilanzierung, da der Gasaustausch sich im Verlauf der letzten Tage deutlich verschlechtert hat. Das FiO musste von 0.4 auf 0.75 gesteigert werden. Nochmal sichten der mikrobiologischen Ergebnisse und gegebenenfalls Umstellung der antibiotischen Therapie. Sollte sich die Patientin jedoch weiter verschlechtern, Eskalation der antibiotischen Therapie auf Meronem und Vanco in Rücksprache mit den Infektiologen. Dann mit Info der Viszeralchirurgen und möglicherweise früherer erneuter Eingriffe. Dann auch ein TEE zum Ausschluss einer Endokardie. Die Angehörigen sind über die Operation und den Verlauf informiert.

In der weiteren Anamnese hat Frau Egli eine Glaukom-OP rechts, eine COPD Gold 2 unter Steroidtherapie, sowie eine mechanische AKE vor drei Jahren, sowie ein Vorhofflimmern.

*Case 4*

Hier liegt Frau Ruth Schüppach, geboren am 16.08.1959. Sie ist 1.73m groß und wiegt 120 kilo. Sie hat vor fünf Tagen eine Bio-AKE bekommen bei Aortenklappenstenose. Am dritten Tag konnte sie auf die Abteilung verlegt werden, ist dann jedoch am vorletzten Abend von der IMC auf die Intensivstation gekommen. Sie zeigte hier ein septisches Zustandsbild. Bei Aufnahme auf die Intensivstation war das CRP 323, die Leukos 23, sowie eine Linksverschiebung von 75%. Wir haben hier mit Tazobac begonnen. Wir haben dann ein pulmonales Katheter eingelegt, hier zeigte sich ein Cardiac-Index von 3.5, eine gemischtvenöse Sauerstoffsättigung von 78% und im TTE zeigte sich der linke Ventrikel hyperdynam. Die rechtsventrikuläre Funktion war eingeschränkt. Der MAP ist so bei 50 mit hochdosierter Noradrenalintherapie gewesen. Das Laktat bei 5.6 mmol. Im Röntgenbild zeigt sich eine leichte Überwässerung, keine gravierende Stauung, keine Infiltrate. Die aktuelle Beatmungseinstellung: Hat sie ein FiO von 0.55, ein PEEP von 10, sowie Tidalvolumina von 350ml. Bei der körperlichen Untersuchung zeigte sich ein instabiles Sternum und eine gerötete Naht. Aufgrund dessen haben die Herzchirurgen dann eine Revision durchgeführt. Hier zeigte sich eine schwere Mediastinitis und ausgerissene Cerclagen. Der Thorax ist weiterhin offen, nur die Haut ist adaptiert. In zwei Tagen soll die erneute Spülung des Mediastinums erfolgen. Die mikrobiologischen Ergebnisse ergaben eine Staph aureus Infektion in den Biopsaten, hier wurde dann auf Floxapen umgestellt. Im Verlauf kam es dann auch zu einem Anstieg der Retentionsparameter, sowie einer rückläufigen Diurese, so dass im Verlauf des Tages mit einer Prismatherapie angefangen wurde. Zudem kam es zum Auftreten eines erneuten Vorhofflimmerns mit ungefähr 110 Schlägen pro Minute, sodass wir hier mit einem Cordarone Perfusor angefangen haben. Die Ernährung wurde begonnen, zur Zeit bekommt die Patientin 1200 kcal und für die Nacht ist eine Kaloriemetrie geplant. Die Angehörigen wurden über das Prozedere informiert.

Die Aufträge für die nächste Schicht sind: Wenn möglich weiter das Noradrenalin reduzieren. Sollte das Noradrenalin gesteigert werden, dann bitte ein erneutes TTE.

In der weiteren Anamnese hat die Patientin ein Hüft-TEP links bei Coxarthrose, ein COPD Gold 3, ein Diabetes Typ 2 und eine PAVK.

*Case 5*

Hier liegt Frau Marlene Bossat, geboren am 06.08.1945. Sie ist 1.59 groß und wiegt 56 kilo. Sie ist vor zwei Tagen out of hospital reanimiert worden, bei initialem Kammerflimmern und einer Downtime von 15 Minuten und einer ROSC nach 34 Minuten. Die Intubation war schwierig vor Ort und es besteht Verdacht auf eine Aspiration. Sie hatte dann nen STEMI im EKG und ist direkt ins Coro gekommen. Dort zeigte sich eine schwere 3-Gefäß-KHK mit dem Verschluss der RIVA. Sie hat da drei Stents bekommen. Ist kreislaufstabil und ohne Katecholamine zu uns auf die Intensivstation gekommen. Der Verlaufs-CK war zweieinhalbtausend, das Trop 400 und das Laktat 2.5 mmol pro Liter. Das Normothermieprotokoll lief bis heute morgen und jetzt ist sie im Aufwachversuch. Sie hat nen GCS von 5 und zeigte hier Myoklonien. Daher haben wir mit Keppra angefangen. Darunter haben sich jedoch die Myoklonien nicht verbessert. Das folgende EEG zeigte schwere Allgemeinveränderungen und einen Verdacht auf einen hypoxischen Hirnschaden. Das MRI vom Schädel soll allenfalls morgen stattfinden. Die Sedation erfolgt jetzt mit Remifentanil, darunter sind die Myoklonien besser. Auch die Ernährung wurde begonnen; die Kaloriemetrie ergab hier 1300 Kilokalorien. Aktuell sind wir aber noch bei Zottennahrung, da die Patientin einen ausgeprägten Reflux hat. Hier haben wir mit Erythromycin gestartet. Die Beatmungseinstellungen sind PEEP 5, FiO2 von 0.45. Im Röntgenbild zeigte sich ein großer Pleuraerguss auf der linken Seite. Den haben wir entlastet und es entleerten sich 850 ml seröser Erguss. Ein Anhalt auf eine Aspiration gab sich im Röntgenbild jedoch nicht. Das TTE ergab ein EF von 45% und wir haben die Herzinsuffizienztherapie mit Captopril 6.25mg alle acht Stunden und Beloc 2 mal 25mg begonnen. Das kann sicherlich noch gesteigert werden.

Die Aufträge für die nächste Schicht: Der Ehemann war mit den Kindern am Bett. Das Gespräch ist noch nicht erfolgt. Das muss jetzt in der laufenden Schicht noch durchgeführt werden, über die eher schlechte Prognose. Dann sollte noch Rücksprache mit den Neurologen gehalten werden, ob wir eine Phenytoinaufsättigung machen oder eine Dosiserhöhung von Keppra durchführen und obs noch ein erneutes EEG braucht.

In der weiteren Anamnese hat die Patientin eine KHK, eine PAVK, eine Diabetes Typ 2 und eine Hypakusis.

*Case 6*

Hier liegt Frau Julia Müller, geboren am 02.03.48. Sie ist 1.63 groß und wiegt 50 Kilo. Sie hat vor fünf Tagen eine in-hospital cardiac arrest erlitten bei initialer Asystolie. Die Downtime betrug fünf Minuten, ROSC hatte sie nach 25 Minuten. Dabei hat sie aspiriert. Im Coro zeigte sich keine KHK. Nun ist sie kreislaufstabil im Sinusrhythmus. Im Aufwachversuch zeigte sich keine Reaktion, im EEG hat sie einen Verdacht auf schwere Schädigung und im MRI zeigte sich ein schwerer hypoxischer Hirnschaden. Der Patient ist ohne Sedation mit einem GCS von 5. Die Beatmungseinstellungen gestalten sich schwierig, das FiO2 beträgt 0.7, der Biox 91%, Plateau-Drücke sind 29 und der PEEP 13. Einen intrinsischen PEEP von 9 hat sie manchmal. Umstellung der Spontanatmung hat noch nicht funktioniert. Darüber hinaus hat die Patientin viel eitriges Sekret abzusaugen, ein TBS ist da abgenommen.

Vor zwei Tagen hat sich die Patientin überbläht und ist fast erneut reanimationspflichtig geworden. Im Röntgenbild zeigt sich eine ausgeprägte Aspirationspneumonie rechts basal. Es wurde mit Augmentin begonnen.

Die enterale Ernährung wurde gestartet, die Laufrate beträgt 800 Kilokalorien pro 24 Stunden. Bei einem erhöhten gastralen Residualvolumen von 1200ml wurde mit Erythrocin und auch Primperan begonnen. Hemodynamisch ist die Patientin stabil, eher ein wenig hyperton. Daher wurde mit Captopril begonnen. Aktuell bekommt die Patientin da 25 mg alle acht Stunden. Bevor mit einem Betablocker begonnen werden soll, muss noch eine TTE durchgeführt werden. Mit dem Ehemann ist besprochen, keine erneute Reanimation. Die Kinder kommen noch vorbei und dann soll morgen ein gemeinsames Gespräch bezüglich der Prognose und der weiteren Behandlungen stattfinden.

Die Aufträge für die nächste Schicht sind Optimierung der Beatmung und Bronchoskopie bei noch sehr viel Sekret, ein TTE zur Beurteilung der EF und den Hausarzt anrufen um mit ihm den mutmaßlichen Patientenwillen zu besprechen. Der Ehemann meinte, dort liegt auch die Patientenverfügung.

*Case 7*

Hier liegt Herr Ueli Moser, geboren am 11.09.72, er ist 1.82 groß und wiegt 90 Kilo. Er liegt bei uns wegen eines Leberversagens bei Leberzirrhose Child C, Status nach C2 Abusus und jetzt ner primär bilären Cholagnitis. Er hat vor fünf Tagen von den Gastroenterologen einen Stent in die Gallengänge bekommen. Hier zeigte sich viel Eiter, er ist dann septisch geworden und zu uns auf die IB gekommen. Weiterhin ist er seitdem hämodynamisch instabil, hat einen MAP von 50mmHg und ist unter hochdosierter Noradrenalintherapie. Der Pulmonaliskatheter zeigt eine gemischtvenöse Sauerstoffsättigung von 80% und einen CardiacIndex von 4. Das maximale Laktat betrug 8.4 mmol. Des Weiteren hat er ein Nierenversagen und ist da seit drei Tagen am Prisma, jedoch ohne Entzug. Die Metabolik ist weiterhin eher schlecht, bei einem Laktat von 5.8 mmol pro Liter. Das Ammoniak von 300 am Anfang ist unter Dufolac- und Xifaxantherapie und im Verlauf sank das Ammoniak auf 146. Ernährt ist der Patient über eine Jejunalsonde mit aktuell 1400ml Sondernahrung. Das aktuelle gastrale Residualvolumen beträgt 1300ml. Hier wurde mit Primperan angefangen um das GRV zu reduzieren. Die antibiotische Therapie besteht aus Meronem und Vancomycin. Das aktuelle CRP ist 279, die Leukozyten betragen 3.9 und das Procalcitonin 12.4. Der Gasaustausch; da hat er ein FiO2 von 0.4, ein PEEP von 10 und die Plateaudrücke sind so um 28. Der Patient hat ab und zu auto-PEEP.

Im Röntgenbild zeigten sich diffuse Verschattungen beidseits und ein Pleuralerguss auf der linken Seite. Den haben wir im Sono bestätigen können, der ist jedoch nicht Punktionswürdig. In der Aszitespunktion fördert der Patient so ca. drei Liter pro 24 Stunden und alle zwei Liter geben wir Albumin. Die Gastroenterologen wollen noch, dass wir mit Terlipressin anfangen und zudem hat der Patient eine massive Gerinnungsstörung, die Thrombozyten sind 13, der INR 4 und die apTT 65.

Die Aufträge für die nächste Schicht sind Albumin wenn's fällig wird, dann Diskussion mit den Gastroenterologen bezüglich des Terlipressins. Dann noch ein Aufwachversuch zur neurologischen Beurteilung und dann Rücksprache mit den Hepatologen und Neurochirurgen bzgl. eines CCTs und ggf. Einlage einer Spiegelbergsonde. Zudem muss die Beatmung noch optimiert werden.

In der weiteren Anamnese hat der Patient Status nach Drogenabusus mit Kokain, THC und Amphetaminen, C2-Konsum, er ist mangelernährt, hat diverse Ulcera in den Unterschenkeln und einen Spritzenabszess in der Leiste vor vier Monaten.

*Case 8*

Hier liegt Frau Katja Bauer, geboren am 10.12.1962, sie ist 1.67 groß und wiegt 75 Kilo. Mittlerweile ist sie seit vier Tagen auf der IB, war davor drei Tage auf der IMC, dann bei Verschlechterung der respiratorischen Situation auf die IB genommen worden. Sie hat eine Pneumonie, die Ätiologie ist hier noch unklar. Im Thorax-CT zeigen sich Milchglastrübungen und konsolidierte Areale in den Unterlappen beidseits, links mehr als rechts. In der mikrobiologischen Diagnostik ist bisher nichts herausgekommen. Eine BAL durch die Pneumologie ist erfolgt. Auch hier keine wegweisenden Resultate: keine Eosinophilie, keine Viren. Zunächst hatten wir mit NIV-Therapie begonnen, dann im Nachtdienst jedoch bei zunehmender Erschöpfung intubiert. Seitdem ist das FiO2 steigend, aktuell 0.9. PEEP 8, Plateaudrücke sind so um 30, das Tidalvolumen beträgt 390ml. Eine Bauchlage hat die Beatmungssituation nicht verbessert. Die antibiotische Therapie erfolgt mit Tazobac seit dem Eintritt. Das CRP ist 275, die Leukozyten sind 18, das Procalcitonin 14, die Stäbchen 40%. Zunächst soll noch keine Umstellung der antibiotischen Therapie erfolgen. Wenn die Patientin dann Fieber bekommt, dann erneute Kultivierung, Katheterwechsel und Rücksprache mit den Infektiologen. Die Sedation erfolgt mit Propofol und Fentanyl am Perfusor, sowie Tracium am Perfusor. Wir haben einen PA-Katheter eingelegt um die Situation besser beurteilen zu können, hier beträgt die gemischtvenöse Sauerstoffsättigung 59%. Das Cardiac-Output 6.7 Liter pro Minute. Die enterale Ernährung ließ sich nicht suffizient aufbauen, sodass die Patientin nun eine kombinierte enterale-parenterale Ernährung erhält.

Die Aufträge für die nächste Schicht: Optimierung der Beatmungssituation, hier soll noch einmal versucht werden, eine Ösophagusdrucksonde einzulegen, das war initial schwierig, da sie sich immer wieder im Mund aufgerollt hat. Bei weiteren Verschlechterungen der Situation: Rücksprache mit dem Betriebsleiter und ggf. Einlage einer venovenösen ECMO.

Die weitere Anamnese der Patientin: Da hat sie einen Status nach Melanom und Resektion, eine Hypothyreose und Status nach Verkehrsunfall vor vier Jahren mit schwerem Schädel-Hirn-Trauma und seitdem hat sie leichte kognitive Einschränkungen.

*English*

*Case 1*

Here lies Mr Reto Stämpfli, born 30.11. 51; 1.78 meters tall, 95 kg; he had a car accident 10 days ago with severe craniocerebral trauma, an intracerebral hemorrhage, initially he had GCS 9, multiple facial skull fractures, as well as a fracture of thoracic vertebrae 4/5 which is unstable; lower leg fractures on both sides, as well as a blunt abdominal trauma with third degree splenic laceration and second degree renal laceration, a thoracic trauma with serial rib fractures on the right. Initially he had an increased intracranial pressure with a maximum of 30. He was then decompressively craniectomised, on the right, on the second day. Afterwards, however, we continued to have problems with intracranial pressure, so initially no attempt was made to wake him up. Now the cerebral pressures are under control; in the wake up attempt test he still has a GCS of 9. The treatment of the facial skull should be done conservatively, as well as the splenic and renal laceration. During the recovery, therapy with the circulatory support medications could be stopped. On the 6th day, there was an increasing deterioration of the ventilatory situation; the X-ray showed shadows on both sides. When the signs of inflammation and fever increased, the antibiotic therapy Augmentin was started. The secretion we were able to aspirate was cloudy-yellow, TBS has been taken and chest sonography showed no pleural effusion. The current ventilation settings are PEEP-8, FiO2= 0.5. In the further recovery, there was a renewed circulatory insufficiency in the sense of a septic shock with postulated pneumonia. Here, the antibiotic therapy was escalated to Tazobac. Under this, the inflammatory parameters decreased, but the retention parameters increased and renal failure occurred. With urea rising to 34 and anuria, as well as hyperkaliemia, continuous renal replacement has now followed for three days. Currently with a withdrawal of 200ml per hour. Last night's calorie shows 2200 kilocalories; enteral nutrition is increased accordingly.

Orders for the next shift: negative balance, waiting for the microbiological diagnosis of pneumonia and, if necessary, termination of the antibiotic therapy. Furthermore, the talk with the partner, Mrs. Gisela Roter, about the further procedure must be held. Here, consent should be obtained for a tracheotomy if the GCS does not improve further; and consultation with the orthopedic surgeons as to when the thoracic vertebrae should be treated.

Mr. Stämpfli's further medical history includes arterial hypertension, status after appendectomy in 1970, status after cataract surgery in 2016 on the left, and status after CVI in 2017 with a slight hemi on the right side.

*Case 2*

Here lies Mrs. Rosi Müller, born on 25.04.1938. She is 1.65 meters tall, she weighs 78 kilos. She fell down a flight of stairs eleven days ago and suffered a traumatic brain injury with intracerebral hemorrhage. Her initial GCS was 6. She was intubated on site, suffered a complex pelvic fracture and a left serial rib fracture, 2-8 ventral. She has a lower leg fracture on the left side, as well as a humerus fracture on the left side. Because of instability, an fixator extern was applied to the pelvis in the emergency room. In the wake up attempt, she initially did not present adequately, with a GCS of 8. The follow-up CT of the head then showed a blooming of the contusion hemorrhage. An EVD was then inserted. It is now open at 15.

On the sixth day, with stable intracranial pressure values, the orthopedic surgeons surgically treated the pelvis. On the wake up attempt, she showed a GCS of 13 and could subsequently be extubated. However, she had to be reintubated because of insufficient respiratory mechanics and a lot of secretions. The current chest X-ray shows atelectasis on the left side. A bronchoscopy was performed. It was possible to remove a lot of glassy, viscous secretion from the middle lobe. After that, the ventilation situation was much better and the FiO2 could be decreased from 0.5 to 0.35. The PEEP is 5. She then developed atrial fibrillation, under which she became hemodynamically unstable and we started norepinephrine. The central venous oxygen saturation was 45. We then floated in a pulmonary catheter. Here the mixed venous saturation was 45, the cardiac index 1.8, so we started with dobutamine. Under this the hemodynamics improved and by means of cardioversion a sinus rhythm could be established and the dobutamine could be reduced. The TTE showed an EF of 45%.

The orders for the next shift: to reduce the dobutamine and noradrenaline, to consult with the thoracic surgeons and to evaluate the unstable thorax and, if necessary, to stabilize the thorax. Then the conversation with the life partner, Mr Reto Schüppach, about the further procedure must take place.

Ms Müller's further medical history includes CHD, paroxysmal atrial fibrillation, status post mamma-ca, hip arthroplasty six years ago on the left side, and hypothyroidism, which is substituted with 150 micrograms of euthyrox.

*Case 3*

Here lies Mrs. Marie Egli, born on 16.07.1946, she is 1.65 meters tall and weighs 69 kilos. She suffered a gastric perforation four days ago and then had an emergency laparotomy with the onset of sepsis. Here she was given a partial gastric resection. Further, in septic shock, she has had high doses of norepinephrine and a positive balance of eight liters. Antibiotic therapy was given with cefepime and metronidazole. Microbiological diagnostics have not shown any evidence of germs to date. In the course of the patient's condition, further circulatory instability occurred, so that we inserted a pulmonary catheter. Echocardiography showed a clearly limited pump function with an LVEF of 30%, so that we started dobutamine. Under this, the circulatory situation improved. Cardiac Index was 1.9, CVD 10, mixed venous oxygen saturation 65% and lactate returned from 8.6 to 3.9 mmol/l. Clinically, the abdomen was clearly tense and the visceral surgeons performed another laparotomy. This revealed a necrotic gastric mucosa, so a complete partial gastric resection was performed. The patient is now two hours out of surgery. The lactate is now 2.5, dobutamine is on a constant dosage, but noradrenaline could be reduced significantly. The third look is scheduled in 48h.

The orders for the next shift are: Reduce norepinephrine if possible, start with negative balancing if periphery is warm and diuresis is maintained as gas exchange has deteriorated significantly over the last few days. The FiO had to be increased from 0.4 to 0.75. Check the microbiological results again and change the antibiotic therapy if necessary. However, if the patient continues to deteriorate, escalation of antibiotic therapy to Meronem and Vanco in consultation with the infectiologist. Then with info from the visceral surgeons and possibly earlier re-interventions. Then also a TEE to rule out endocarditis. The relatives are informed about the operation and the course.

In the further medical history, Mrs. Egli has a glaucoma operation on the right, a COPD Gold 2 under steroid therapy, as well as a mechanical aortic valve replacement three years ago, and atrial fibrillation.

*Case 4*

Here lies Ruth Schüppach, born on 16.08.1959. She is 1.73m tall and weighs 120 kilos. She had a bio-aortic valve replacement five days ago for aortic valve stenosis. On the third day she was transferred to the ward, but on the evening before last she was transferred from the IMC to the intensive care unit. She presented with a septic condition. On admission to the intensive care unit, the CRP was 323, the leukocytes 23, and a left shift of 75%. We started Tazobac here. We then inserted a pulmonary catheter, this showed a Cardiac Index of 3.5, mixed venous oxygen saturation of 78% and TTE showed the left ventricle hyperdynamic. Right ventricular function was impaired. MAP has been so at 50 with high dose norepinephrine therapy. The lactate was 5.6 mmol. X-ray shows mild hyperhydration, no serious congestion, no infiltrates. Current ventilatory setting: she has a FiO of 0.55, a PEEP of 10, and tidal volumes of 350ml. Physical examination revealed an unstable sternum and a reddened suture. Due to this, the cardiac surgeons then performed a revision. This revealed severe mediastinitis and avulsed cerclages. The thorax is still open, only the skin is adapted. The mediastinum is to be rinsed again in two days. The microbiological results revealed a Staph aureus infection in the biopsies, which were then switched to Floxapen. In the course of the day, there was an increase in the retention parameters and a decrease in diuresis, so that Prisma therapy was started in the course of the day. In addition, atrial fibrillation reappeared with about 110 beats per minute, so we started with a Cordarone perfusor. Nutrition was started, at the moment the patient gets 1200 kcal and a calorimetry is planned for the night. The relatives have been informed about the procedure.

The orders for the next shift are: Continue to reduce norepinephrine if possible. If the norepinephrine is increased, then please have another TTE.

In the further history, the patient has a left hip TEP for coxarthrosis, a COPD Gold 3, a diabetes type 2 and a PAD.

*Case 5*

Here lies Mrs. Marlene Bossat, born on 06.08.1945. She is 1.59 meters tall and weighs 56 kilos. She was resuscitated out of hospital two days ago, with initial ventricular fibrillation and a downtime of 15 minutes and a ROSC after 34 minutes. On site intubation was difficult and aspiration was suspected. She had a STEMI in the ECG and went straight to the coro. There she had a severe 3-vessel CHD with occlusion of the RIVA. She received three stents. She was admitted to the intensive care unit in stable condition and without catecholamines. The course CK was two and a half thousand, the trop 400 and the lactate 2.5 mmol per liter. The normothermia protocol ran until this morning and now she is in recovery. She has a GCS of 5 and showed myocloni here. Therefore we started with Keppra. However, the myocloni did not improve under this treatment. The following EEG showed severe general changes and a suspicion of hypoxic brain damage. The MRI of the brain will probably take place tomorrow. Sedation is now with remifentanil, under which the myocloni are better. Nutrition has also been started; calorimetry showed 1300 kilocalories. Currently, however, we are still on villous feeding, as the patient has a pronounced reflux. Here we started with erythromycin. The ventilation settings are PEEP 5, FiO2 of 0.45. The X-ray showed a large pleural effusion on the left side. We relieved it and 850 ml of serous effusion emptied. However, there was no indication of aspiration in the X-ray. The TTE showed an EF of 45% and we started heart failure therapy with captopril 6.25mg every eight hours and Beloc 2 times 25mg. This can certainly be increased.

Orders for the next shift: husband was at the bedside with the children. The conversation has not been done yet. This still needs to be done now in the current shift, about the rather poor prognosis. Then there should still be consultation with the neurologists as to whether we should do a phenytoin resaturation or a dose increase of Keppra and whether we still need another EEG.

In the further history, the patient has a CHD, a PAD, a type 2 diabetes and a hypoacusis.

*Case 6*

Here lies Mrs. Julia Müller, born on 02.03.48. She is 1.63 meters tall and weighs 50 kilos. Five days ago she suffered an in-hospital cardiac arrest with initial asystole. The downtime was five minutes, she had ROSC after 25 minutes. She aspirated in the process. Coro showed no CHD. Now she is stable in sinus rhythm. The wake-up test showed no response, EEG has suspected severe damage and MRI showed severe hypoxic brain damage. The patient is without sedation with a GCS of 5. Ventilation settings are difficult, FiO2 is 0.7, Biox 91%, plateau pressures are 29 and PEEP 13. She sometimes has an intrinsic PEEP of 9. Switching to spontaneous breathing has not worked yet. In addition, the patient has a lot of purulent secretions to aspirate, a TBS has been taken off.

Two days ago, the patient overinflated and almost required resuscitation again. The X-ray shows marked aspiration pneumonia on the right basal side. Augmentin was started.

Enteral nutrition was started, running at 800 kilocalories per 24 hours. With an increased gastric residual volume of 1200ml, erythrocin was started and also Primperane. Hemodynamically the patient is stable, rather a little hypertensive. Therefore, Captopril was started. The patient currently receives 25 mg every eight hours. Before starting a beta-blocker, a TTE has to be performed. It has been agreed with the husband not to reanimate again. The children are still coming round and then a joint discussion is to take place tomorrow regarding the prognosis and further treatments.

The orders for the next shift are to optimize ventilation and bronchoscopy as she still has a lot of secretion, a TTE to assess the EF and to call the GP to discuss the presumed will of the patient. The husband said that the living will is also there.

*Case 7*

Here lies Mr. Ueli Moser, born on 11.09.72, he is 1.82 meters tall and weighs 90 kilos. He is with us because of liver failure with liver cirrhosis CHILD C, status after C2 abuse and now primary biliary cholagitis. Five days ago, the gastroenterologists put a stent in his bile ducts. This showed a lot of pus, he then became septic and came to us in the ICU. Furthermore, he has been hemodynamically unstable since then, has a MAP of 50mmHg and is on high-dose noradrenaline therapy. The pulmonary catheter shows a mixed venous oxygen saturation of 80% and a Cardiac Index of 4. The maximum lactate was 8.4 mmol. Furthermore, he has renal failure and has been on Prisma for three days, but without withdrawal. The metabolic rate is still rather poor, with a lactate of 5.8 mmol per liter. The ammonia of 300 at the beginning is under Dufolac and Xifaxan therapy and in the course of time the ammonia decreased to 146. The patient is fed via a jejunal tube with currently 1400ml special food. The current gastric residual volume is 1300ml. Primperan was started to reduce the GRV. Antibiotic therapy consists of Meronem and Vancomycin. Current CRP is 279, leukocytes are 3.9 and procalcitonin is 12.4. Gas exchange; there he has a FiO2 of 0.4, PEEP of 10 and plateau pressures are around 28. The patient has auto-PEEP every now and then.

The X-ray showed diffuse shading on both sides and a pleural effusion on the left side. We were able to confirm this on sonography, but it is not worthy of puncture. In the ascites puncture, the patient produces about three liters per 24 hours and we give albumin every two liters. The gastroenterologists still want us to start with Terlipressin and in addition the patient has a massive coagulation disorder, the platelets are 13, the INR 4 and the apTT 65.

The orders for the next shift are albumin when it's due, then discussion with the gastroenterologists regarding the terlipressin. Then another wake-up test for neurological assessment and then consultation with the hepatologists and neurosurgeons regarding a CCT and insertion of a Spiegelberg probe if necessary. In addition, the ventilation must still be optimized.

In the further history, the patient has a status of drug abuse with cocaine, THC and amphetamines, C2 consumption, he is malnourished, has various ulcers in the lower legs and a syringe abscess in the groin four months ago.

*Case 8*

Here lies Mrs. Katja Bauer, born on 10 December 1962, she is 1.67 meters tall and weighs 75 kilos. She has now been on the ICU for four days, before that she was on the IMC for three days, she was taken to the ICU when her respiratory situation worsened. She has pneumonia, the aetiology of which is still unclear. The chest CT shows milk glass opacities and consolidated areas in the lower lobes on both sides, more on the left than on the right. Microbiological diagnostics have not yet revealed anything. A BAL was performed by the pneumologist. Again, no conclusive results: no eosinophilia, no viruses. Initially, we started with NIV therapy, but then intubated him during the night shift when he became increasingly exhausted. Since then, FiO2 has been increasing, currently 0.9. PEEP 8, plateau pressures are around 30, tidal volume is 390ml. Prone position has not improved the ventilatory situation. Antibiotic therapy has been given with Tazobac since admission. The CRP is 275, the leucocytes are 18, the procalcitonin 14, the rods 40%. Initially, there should be no change in antibiotic therapy. If the patient then develops a fever, then renewed cultivation, change catheter and consultation with the infectiologists. Sedation is done with propofol and fentanyl on the perfusor, and tracium on the perfusor. We have inserted a PA catheter to better assess the situation, here the mixed venous oxygen saturation is 59%. The cardiac output is 6.7 liters per minute. The enteral nutrition could not be established sufficiently, so that the patient now receives a combined enteral-parenteral nutrition.

The orders for the next shift: Optimization of the ventilation situation, here another attempt should be made to insert an esophageal pressure tube, which was initially difficult because it kept rolling up in the mouth. In case of further deterioration of the situation: Consultation with the operation manager and, if necessary, insertion of a venovenous ECMO.

The patient's further medical history: She has a status after melanoma and resection, hypothyroidism and status after a traffic accident four years ago with severe craniocerebral trauma, and since then she has had slight cognitive impairments.

## Part 2: Full Demographic Questionnaire

*German*

Additional Questions: Individual Variables

|  |
| --- |
| Wie gestresst waren Sie in der letzten Woche? |
| Wie gut schätzen Sie Ihre Hörleistung ein? |
| Wie hoch ist ihre Konzentrationsfähigkeit in lärmigen Umgebungen? |
| Wie hoch schätzen Sie Ihre Konzentrationsfähigkeit heute ein? |
| Wie schätzen Sie ihre Energie während der Studie heute ein? |
| Wie motiviert waren Sie eine gute Leistung während der Studien zu erbringen? |
| Haben Sie sich heute Notizen so gemacht, wie auch während gewohnten Visiten? |

*0 50 100*

*ja nein*

*English*

Additional Questions: Individual Variables

|  |
| --- |
| How stressed did you feel the past week? |
| How well do you hear? |
| How high is your ability to concentrate in noisy environments? |
| How high do you rate your ability to concentrate today? |
| How do you rate your energy during the study today? |
| How motivated were you to perform well during the study? |
| Did you take your notes in the same way as during habitual ward rounds? |

*0 50 100*

*yes no*

## Part 3: Ward round questionnaire

*German*

*Case 1*

1. Ist die WS-Fraktur stabil?

2. Welchen Grad hat die Nierenlazeration?

3. Wie wurde der erhöhte intrakranielle Druck behandelt?

4. Wird Herr Stämpli aktuell mit Kreislaufunterstützenden Medikamenten behandelt?

5. Welche antibiotische Therapie läuft aktuell?

6. Welcher Keim wird behandelt?

7. Für welche Intervention soll die Einwilligung eingeholt werden und unter welcher Bedingung würde sie durchgeführt werden?

8. Was muss beim Aufwachversuch aufgrund der Anamnese beachtet werden?

*Case 2*

1. Wie war der initial GCS?

2. Welche Rippen sind gebrochen?

3. Wie ist die EVD eingestellt?

4. Wie war der GCS im letzten Aufwachversuch?

5. Warum dekompensiert der Kreislauf der Patientin?

6. Mit welchen Medikamenten erfolgt die Kreislaufunterstützung?

7. Welche EF zeigt sich im TTE?

8. Was soll mit dem Ehemann besprochen werden?

*Case 3*

1. Welchen Keim ergab die mirkobiologische Diagnostik?

2. Welche EF zeigte sich im TTE?

3. Was ist das aktuelle Laktat?

4. Wann ist der 3rd look geplant?

5. Wie ist das aktuelle FiO2?

6. Mit welchen Medikamenten soll die Antibiose eskaliert werden?

7. Welche weiterführenden diagnostischen Massnahmen müssen bei einer Verschlechterung durchgeführt werden?

8. Sind die Angehörigen über die Operation informiert worden?

*Case 4*

1. Wieso wurde sie wieder von der IMC zurückverlegt?

2. Wie ist die LV-Funktion?

3. Wie hoch ist das FiO2?

4. Wann soll die Revision erfolgen?

5. Was ergab die mikrobiologische Diagnostik?

6. Wie ist die Nierenfunktion der Patientin?

7. Sind die Angehörigen informiert worden?

8. Wie lauten die Aufträge für die nächste Schicht?

*Case 5*

1. Wie lange war die Downtime?

2. Wann trat der ROSC ein?

3. Was war der Grund für die Reanimation?

4. Welcher Befund zeigt sich in der Koronarangiographie?

5. Welcher GCS bot sich beim initialen Aufwachversuch?

6. Ist der Patient gemäss EEG im Status epileptius?

7. Weshalb haben sich die Myoklonien verbessert?

8. Was soll mit den Angehörigen besprochen werden?

*Case 6*

1. Wo fand die Reanimation statt?

2. Wie war die Downtime?

3. Was zeigen die neurologischen Untersuchungen (gemäss MRI)?

4. Wieso musste die Patientin beinahe erneut reanimiert werden?

5. Was zeigt das Röntgenbild vom Thorax?

6. Mit wem soll das weitere Procedere besprochen werden?

7. Was sind die Aufträge für die nächste Schicht?

8. Wie viele Kalorien wurden der Patientin enteral zugeführt?

*Case 7*

1. Wie wird die hämodynamische Situation eingeschätzt?

2. Wie hoch ist der aktuelle Laktat-Wert?

3. Wie ist der aktuelle Ammoniak-Wert?

4. Über welche Sonde wird der Patient ernährt?

5. Wie viel Sondennahrung bekommt der Patient?

6. Welche antibiotische Therapie läuft aktuell?

7. Was ist der Therapieplan der Gastroenterologen?

8. Mit welchen Fachdisziplinen muss bzgl. des weiteren Prozederes geredet werden?

*Case 8*

1. Wie lange ist die Patientin bereits im Spital?

2. Was zeigte das Thorax CT ?

3. Seit wann wird sie antibiotisch behandelt?

4. Wie ist das aktuelle Fi02?

5. Welche Schritte sollen bei allfälligem Auffiebern unternommen werden ?

6. Wie erfolgt die Analgo-Sedation und Relaxation?

7. Was soll bei einer Verschlechterung ggf. eingebaut werden?

8. Ursache der leichten kognitiven Einschränkung?

*English*

*Case 1*

1. Is the vertebral fracture stable?

2. What is the degree of renal laceration?

3. How was the increased intracranial pressure treated?

4. Is the patient currently being treated with circulation-supporting medication?

5. What antibiotic therapy is currently in progress?

6 Which bacteria is being treated?

7. For what intervention should consent be obtained and under what conditions would it be given?

8. What must be taken into account when trying to wake up based on the anamnesis?

*Case 2*

1. How was the initial GCS?

2. Which ribs are broken?

3. How is the EVD adjusted?

4. How was the GCS in the last wake-up attempt?

5. Why did the patient's circulation decompensated?

6. Which drugs are used to support the circulation?

7. What EF is shown in the TTE?

8. What should be discussed with the husband?

*Case 3*

1. What bacteria did the microbiological diagnostics reveal?

2 Which EF was shown in the TTE?

3 What is the current lactate level?

4. When is the 3rd look planned?

5. What is the current FiO2?

6. Which drugs should be used to help improve the treatment with antibiotics?

7. What further diagnostic measures must be carried out if the condition deteriorates?

8. Have the relatives been informed about the operation?

*Case 4*

1. Why was she transferred back from the IMC?

2. How is the left ventricle (LV) function?

3. How high is the fraction of inspired oxygen (FiO2)?

4. When should the revision take place?

5. What did the microbiological diagnostics show?

6. What is the patient's renal function?

7. Have the relatives been informed?

8. What are the assignments for the next shift?

*Case 5*

1. How long was the downtime?

2. When did the ROSC occur?

3. What was the reason for the resuscitation?

4 What is the result of the coronary angiography?

5. What GCS was present at the initial wake-up attempt?

6. Is the patient in epileptic status according to the EEG?

7. Why have the myocloni improved?

8. What should be discussed with the relatives?

*Case 6*

1. Where did the resuscitation take place?

2. How long was the downtime?

3. What do the neurological examinations (according to the magnetic resonance imaging (MRI)) show?

4 Why did the patient almost have to be resuscitated?

5 What does the chest x-ray show?

6. With whom should the further procedure be discussed?

7. What are the orders for the next shift?

8. How many calories were administered enterally to the patient?

*Case 7*

1. How is the hemodynamic situation assessed?

2. What is the current lactate level?

3. What is the current ammonia value?

4. Which tube is used to feed the patient?

5. How much parenteral nutrition does the patient receive?

6. What antibiotic therapy is currently in progress?

7. What is the gastroenterologists' treatment plan?

8. Which specialist should be consulted regarding further procedures?

*Case 8*

1. How long has the patient been in hospital?

2. What did the chest computer tomography (CT) show?

3. Since when has she been receiving antibiotic treatment?

4. What is the current Fi02 level?

5. What steps should be taken in case of fever?

6. How is the analog sedation and relaxation performed?

7. What should possibly be incorporated if there is any deterioration?

8. Cause of the slight cognitive impairment?

## Part 4: Audio files

The audio files were played at a sound pressure level of 70 dB SPL. The sample rate of the files was left at 44.1 kHz. The mixtures (background sound and recital of the ward round) have 100ms rise/fall times. All the audio files were normalized at -23 Loudness Units relative to Full Scale (LUFS), as recommended in EBU R 128-2014. In order to present the generated files at the required signal level of 70 dB SPL, a pink noise was used as it approximates the speech spectrum [1]. The normalized noise was used to set the level to 70 dB SPL.

In a pre-study with 23 participants (23-76 years) the ideal difference between the speaking voice and the background noise of -5 dBA was investigated. The aim was to reassure the understanding of the speaking voice, despite the background noise. The study participants rated their hearing performance on a five-point Likert scale (1 “very bad” to 5 “perfect”) and participants who rated their hearing performance <3 (“neither good nor bad”) were excluded from the study. The remaining 21 participants listened on their private computer to an eleven second (+ 1.2 seconds) audio excerpt from each case and then indicated the number of words they did not understand on a clearly visible button. The volume was decided by the study participants. They were instructed to choose their preferred volume on an example audio excerpt and to not change it during the testing. The excerpts were played on a sound to noise ratio (SNR) of 0, -2, -4 and -6 dBA. The excerpts were randomized so that all participants listened to four excerpts of every case with another SNR (in total 8 cases x 4 excerpts = 32 excerpts per participants). The results are displayed in Table 1.

*Table 1*

*Percentage of not understood words.*

| **SNR Level** | 0 (No Noise) | -2 dB(A) | -4 dB(A) | -6 dB(A) |
| --- | --- | --- | --- | --- |
| **Incomprehensibility** | 0.14% | 3.57% | 4.79% | 7.07% |

*Note.:* Sound to noise ratio = SNR

Bradlow et al. (1996) [2] found an intelligibility of 81.1% - 93.4% for normal speech. Meaning, that people who understand speech very well, do not understand 6.6% of the words. Since in the main study the participants will have context, which helps in understanding speech [3], our assumption is, that the participants will understand the ward round audio rather well. Therefore, a SNR -5 dB(A) was chosen, which is between 4.79 and 7.07% incomprehensibility from the pretest (Table 1) which approximately corresponds to the 6.6% determined to be normal.

*References*

[1] Voss RF, Clarke J. ‘1/fnoise’ in music and speech. Nature 1975;258:317–8. https://doi.org/10.1038/258317a0.

[2] Bradlow AR, Torretta GM, Pisoni DB. Intelligibility of normal speech I: Global and fine-grained acoustic-phonetic talker characteristics. Speech Communication 1996;20:255–72. https://doi.org/10.1016/S0167-6393(96)00063-5.

[3] Pollack I, Pickett JM. Intelligibility of excerpts from fluent speech: Auditory vs. structural context. Journal of Verbal Learning and Verbal Behavior 1964;3:79–84. https://doi.org/10.1016/S0022-5371(64)80062-1.
